# Acute Bright's Disease Simulated by Renal Calculus

**Published:** 1907-10-19

**Authors:** 


### Illustrative Cases.

### ACUTE BRIGHT'S DISEASE SIMULATED BY RENAL CALCULUS.

The following case illustrates two points—namely:—

1. That renal calculus may exist for years, long enough to convert a kidney into a functionless hydro-nephrotic sac, without causing any colic or other symptoms suggesting the presence of a stone.

2. That the effects of chronic ascending nephritis may be precisely similar to those of ordinary Bright's disease.

The diagnosis of acute or chronic Bright's disease seemed to be so clear that the finding of a calculus and hydro-nephrosis at the autopsy was an absolute surprise.

The patient was a man aged 41, who had both acute rheumatism and scarlet fever as a youth, but recovered completely from both, and remained in perfect health until sixteen months before his death. At no time had he complained of abdominal pain of any kind, still less of anything so severe as colic. He never had hæmaturia. His first trouble, sixteen months before he died, was epistaxis, which came on spontaneously, and was so persistent that, in addition to absolute rest flat in bed, it was necessary to plug the nares. Upon examination he was found to have a considerably hypertrophied but compensated heart, a high blood-pressure, and albuminuria with casts. There was no œdema at this time and the diagnosis was epistaxis from chronic nephritis. There was no albuminuric retinitis.

He recovered from the nose-bleeding in a day or two, and remained quite well in his general health for thirteen months; that is to say, until three months before he died. He then began to feel weak and short of breath, and had to take to his bed. He suffered from severe headache, was puffy under the eyes, and had slight œdema of the ankles. The cardiac impulse was in the sixth left intercostal space, half an inch outside the nipple line; there was no bruit, but the first sound at the impulse was prolonged and the second sound in the aortic area was greatly accentuated. The lungs were natural. The urine amounted to 40 to 50 ounces a day, had a specific gravity of 1012, and contained four to five parts per thousand of albumen; microscopically, red corpuscles, renal epithelial cells, epithelial, granular, and hyaline tube casts were found, and there were no pus corpuscles nor any crystals. He improved a little for a month, remaining in bed all the time; his most troublesome symptom was headache, which was bad enough to prevent his sleeping. The albumen, blood, and casts in his urine all persisted, and presently he began to get extensive œdema of ankles, legs, thighs, back, penis, and scrotum. It seemed obvious that he was suffering from a typical acute exacerbation of a chronic tubal nephritis. He gradually got weaker and weaker, and more water-logged; finally his heart dilated, and he died of chronic uræmia and cardiac failure.

At the autopsy the subcutaneous tissues of his trunk and lower limbs were found waterlogged. The lungs were partially compressed by the large heart, and though there was no pleurisy, there were ten ounces of clear serous fluid in the right pleural cavity, and eight ounces in the left. There was no pericarditis. The heart was big, weighing sixteen ounces, the hypertrophy affecting mainly the left ventricle; there had been a terminal dilatation of the mitral ring, leading to regurgitation, and consequently to considerable dilatation and moderate hypertrophy of the left auricle, right ventricle, and right auricle; the valves were healthy, the coronary arteries good, and the muscle sound. The aorta and arteries generally were free from atheroma or arterio-sclerosis. The liver was enlarged and nutmegged from the terminal heart failure; and the spleen was firm, dark, and cardiac. The kidneys were the most abnormal organs in the body. The right was a large multi-locular hydronephrotic sac filled with fluid that was unable to escape; before it was cut open, the sac, with its contained fluid, weighed 33 ounces; each calyx, with its corresponding medulla and cortex, was overdistended with clear, colourless liquid, and no kidney substance was left at all; the cause of this was an antlered calculus of oxalate of lime which filled the renal pelvis and, by reason of a small projecting knob which was firmly engaged in the outlet, completely obstructed the ureter. The stone was prevented from shifting its position by several bands and bridles of fibrous tissue, which kept it firmly bound in one situation. The left kidney weighed 6½ ounces, and had apparently hypertrophied by way of compensation for the right. It was firm and cyanosed from heart failure; the capsule peeled readily, leaving a good smooth cortex free from cysts; the arterioles were not thickened, but there was a blotchy appearance at the junction of cortex and medulla in some places, as though from focal acute nephritis. There was a small calculus in the pelvis of this kidney also, but no hydronephrosis. Sections of the renal tissue were examined microscopically, and they showed a mixed condition of chronic and acute ascending nephritis. There were some areas in which both tubules and glomeruli were natural; others where there was excess of mature interstitial fibrous tissue, with atrophy of many of the glomeruli; and others again where there were collections of small round cells in the interstitial tissue, not far enough advanced to constitute abscesses, but surrounded by tubules in which there was cloudy swelling and catarrh of the epithelium.

The rest of the genito-urinary organs were healthy; it seemed clear that the renal calculi had first caused chronic renal fibrosis, which was the cause of the hypertrophied heart, high blood-pressure, and epistaxis; and that, when the right kidney had become functionless, owing to complete atrophy of its substance and its conversion into a hydro-nephrotic bag, an acute ascending nephritis had developed in the left kidney; this and heart failure together being the cause of death, which was therefore indirectly due to the unsuspected renal calculi.

